# Genome-Wide Identification and Expression Analysis of LBD Gene Family in *Neolamarckia cadamba*

**DOI:** 10.3390/ijms27020693

**Published:** 2026-01-09

**Authors:** Chuqing Cai, Linhan Tang, Guichen Jian, Qiuyan Qin, Huan Fan, Jianxia Zhang, Changcao Peng, Xiaolan Zhao, Jianmei Long

**Affiliations:** Guangdong Key Laboratory for Innovative Development and Utilization of Forest Plant Germplasm, College of Forestry and Landscape Architecture, South China Agricultural University, Guangzhou 510642, China; caichuqing1224@163.com (C.C.); tanglinhan0721@163.com (L.T.); gustave017@163.com (G.J.); 15118568622@163.com (Q.Q.); fanh240228@163.com (H.F.); zhangjianxia@scau.edu.cn (J.Z.); ccpeng@scau.edu.cn (C.P.); xiaolanpeng@scau.edu.cn (X.Z.)

**Keywords:** *LBD* gene family, *Neolamarckia cadamba*, expression pattern, abiotic stress

## Abstract

Lateral Organ Boundaries Domain (LBD) proteins are plant-specific transcription factors characterized by a typical N-terminal LOB domain and are critical for plant growth, development, and stress response. Currently, LBD genes have been investigated in various plant species, but they have yet to be identified in *Neolamarckia cadamba*, known as a ‘miracle tree’ for its fast growth and acknowledged for its potential medicinal value in tropical and subtropical areas of Asia. In this study, a total of 65 *NcLBD* members were identified in *N. cadamba* by whole-genome bioinformatics analysis. Phylogenetic analysis revealed their classification into two clades with seven distinct groups, and their uneven distribution across 18 chromosomes, along with 6 tandem repeats and 58 segmental duplications. Furthermore, enrichment analysis of transcription factor binding motifs within *NcLBD* promoters identified the MYB-related and WRKY families exhibited the most significant enrichment in the *NcLBD* promoter. Protein interaction network analysis revealed potential interactions among NcLBD proteins, as well as their interactions with various transcription factors. RNA-seq and qRT-PCR analyses of *NcLBDs* transcript levels showed distinct expression patterns both across various tissues and under different hormone and abiotic stress conditions. Specifically, *NcLBD3*, *NcLBD37*, and *NcLBD47* were highly expressed in vascular cells and induced by abiotic stress, including cold, drought, and salt, suggesting their significant role in the processes. In summary, our genome-wide analysis comprehensively identified and characterized LBD gene family in *N. cadamba*, laying a solid foundation for further elucidating the biological functions of *NcLBD* genes.

## 1. Introduction

The Lateral Organ Boundaries Domain (LBD) protein belongs to a plant-specific transcription factor family characterized by a conserved Lateral Organ Boundaries (LOB) domain in N-terminal region. The LOB domain is composed of three parts, including a zinc finger-like motif, a GAS block (Gly-Ala-Ser), and a leucine chain-like coiled helical motif. Based on the characteristics of LOB domain, the LBD family can be classified into two major groups, designated as Class I and Class II. Class I contains a highly conserved zinc finger-like CX2CX6CX3C motif (where C represents a conserved cysteine residue and X represents an unconserved amino acid residue) and a GAS-block region. It generally starts with the FX2VH sequence and ends with the DP(V/I)YG sequence, containing 49 amino acids. An LX6LX3LX6L helical coiled structure is similar to a leucine zipper, which may be related to protein dimerization. Class II contains only one conservative structure motif similar to zinc finger. The majority of LBD family members belonged to Class I and played essential roles in plant organ development and signaling transduction [1]. In contrast, Class II LBD proteins primarily participated in biosynthesis of secondary metabolites in plants [2]. Since the discovery of 43 LBD genes in *Arabidopsis thaliana* [3], extensive sequencing of plant genomes has uncovered the presence of LBD transcription factor family members across a variety of species, including 37 in sour jujube [4], 67 in apple [5], 55 in poplar [6], and 47 in common bean [1].

Previous studies have shown that the LBD family members demonstrate functional conservation in the regulation of root development across various plant species. For instance, Arabidopsis transcription factors *ARF7* and *ARF19* could directly bind to the promoter regions of *AtLBD16* and *AtLBD29*, regulating lateral root formation by activating the expression of these genes [7]. The PgARF-PgLBD module also mediated auxin signaling to regulate lateral root development in *Pananx ginseng* [8]. *OsWOX11* interacted with histone demethylase JMJ7706 to activate *OsLBD16*, jointly regulating crown root development in rice [9]. In addition, LBD proteins also play a crucial role in plant leaves and floral organ development. Ectopic expression of *AtLOB* led to alterations in the size and shape of leaves and floral organs, and caused both male and female sterility. The *lbd10* mutant exhibited abnormal development in 10–15% of the pollen, while the *lbd27* mutants showed a 70% rate of pollen abortion, and all pollen was aborted and severely shriveled in the *lbd10 lbd27* double mutants, indicating that *AtLBD10* and *AtLBD27* were crucial for pollen development [10]. Overexpression of apple *MdLBD11* led to phenotypic alterations, including upward curling leaves, delayed flowering, downward-pointing flowers, siliques, and other abnormal traits [11]. The LBD family also plays a crucial role in regulating secondary growth in plants. In *Eucalyptus grandis*, *EgLBD37* significantly promoted secondary xylem formation, while *EgLBD29* substantially increased phloem fiber yield [12]. The LBD11-ROS feedback regulatory pathway modulated meristem proliferation and secondary growth in *A. thaliana* [13]. *PagLBD21* acted as an inhibitor of secondary xylem development in poplar [14]. Overexpression of *PagLBD3* thickened poplar stem segments, and *PagLBD3* also interacted with *PagWOX4* to mediate regulation of vascular tissue development in poplar [15].

Furthermore, the LBD genes are crucial in hormone signaling pathways and in facilitating stress responses across various plant species. Ninety LBD homologous genes had been characterized in soybean, wherein *GmLBD12* exhibited responsiveness to various abiotic stresses (drought, salt, cold) and phytohormones including auxin (indole acetic acid, IAA), abscisic acid (ABA), and salicylic acid (SA) treatment [16]. *AtLBD15* could enhance tolerance of water-deficit stress and ABA sensitivity. In addition, drought stress-induced ABA promoted the expression of *AtLBD15*, which directly activated the expression of *AtABI4*, thereby leading to stomatal closure in *A. thaliana* [17]. Knockout of *SlLBD40*, which played a role in jasmonic acid signaling, enhanced drought tolerance in tomato, indicating its negative regulator in drought tolerance [18]. The expression of *VvLBD01*, *VvLBD02*, *VvLBD04*, *VvLBD08,* and *VvLBD18* in grape was associated with their responses to treatments involving sodium chloride (NaCl), mannitol, heat stress, and low temperature [19]. Under ABA treatment, the orchid genes *CsiLBD13*, *CsiLBD19,* and *CsiLBD21* exhibited tissue-specific expression changes [20].

*Neolamarckia cadamba* is an important timber tree in southern Asia and is famous for being a ‘miracle tree’ due to its super rapid growth. The LBD gene family has been identified and investigated in numerous species, such as *Arabidopsis* [3], polyploid wheat [21], rice [22], *Pinus massoniana* [23], poplar [6], and apple [5], but it has yet to be identified in the *N. cadamba*. The availability of the *N. cadamba* genome sequence enables the comprehensive identification of all LBD genes in this species. Vascular development is a key biological process in woody plants and serves as the foundation for wood formation. *N. cadamba* thrives in moist environments with relatively high temperatures (20–24 °C), thereby limiting its potential geographic range [24]. Numerous studies have demonstrated that LBD genes play crucial roles not only in vascular development but also in response to cold and drought stress. However, the specific LBD genes involved in these processes in *N. cadamba* remained unidentified. In the present study, bioinformatics methods were used to identify the LBD gene family, and the classification, gene structure, chromosome localization, synteny, conserved motifs, enrichment analysis of transcription factor binding motifs on the *NcLBD* promoters, protein interaction network, and gene expression were explored in systematical analysis. Our findings lay a theoretical foundation for in-depth study of the functions of *LBD* genes in vascular development and cold and drought stress response in *N. cadamba*.

## 2. Results

### 2.1. Identification and Phylogenetic Analysis of NcLBD Genes

A total of 65 LBD family members were identified in *N. cadamba* using the combination of BlastP and HMMER alignment search. They were subsequently named as *NcLBD1-NcLBD63*, *NcLOB1,* and *NcLOB2* according to their location on chromosomes and homology with LBD family members of *A. thaliana*. The amino acid lengths of all the identified *NcLBDs* ranged from 122 to 823, of which *NcLBD9* was the longest protein and *NcLBD17* was the shortest protein. The molecular weight (MW) of *NcLBDs* varied from 13.89 to 91.94 kDa, with the isoelectric point (pI) ranging from 4.14 to 9.41. Interestingly, the pI of 35 *NcLBDs* was greater than 7.0, while 40 *NcLBDs* was less than 7.0. Instability index of *NcLBDs* ranged from 24.7 to 80.02, and the aliphatic index was between 56.18 and 91.72. The grand average of hydropathy of *NcLBDs* was between −0.738 and −0.02, indicating that they were all hydrophilic proteins (Table S1).

**Figure 1:**
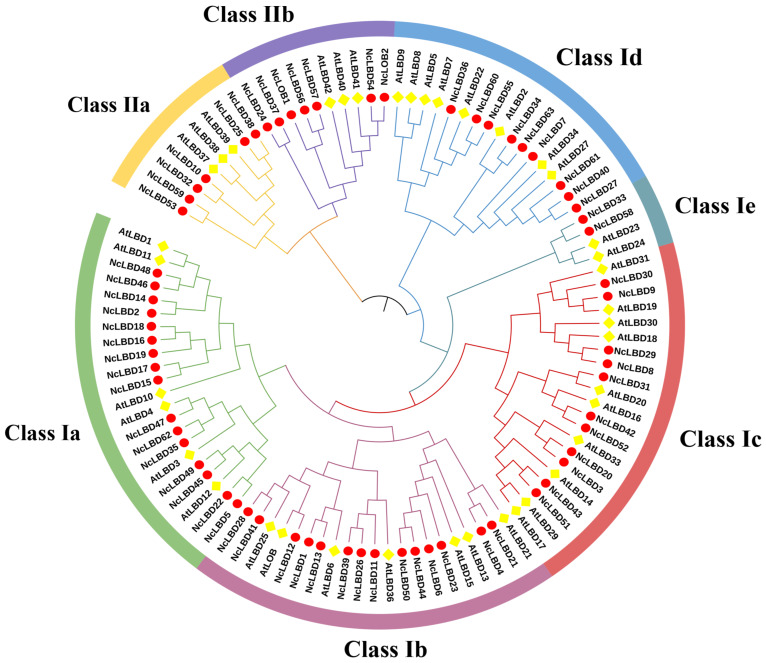
Phylogenetic tree representing relationships among LBD proteins of *N. cadamba* and *A. thaliana*. A phylogenetic tree was constructed for 65 NcLBD and 43 AtLBD proteins with 1000 bootstrap replicates using maximum likelihood (ML) method. The subfamilies of LBD proteins, Class Ia-Ie, IIa, and IIb, are marked with different colored arcs. LBD proteins from *N. cadamba* and *A. thaliana* are signed with the red and yellow circles, respectively. Nc: *N. cadamba*; At: *A. thaliana*.

To enhance our understanding of the phylogenetic relationship among LBD proteins, a phylogenetic tree was constructed based on LBD proteins from *N. cadamba* and *A. thaliana* using maximum likelihood (ML) method. As shown in Figure 1, all the LBD proteins were divided into two clades according to the classification established in a previous study on *A. thaliana* [3], suggesting an evolutionary conservation between these two species. Class I consisted of 52 *NcLBD* members, which could be further divided into five subgroups, with sixteen, fourteen, eleven, two, and nine members belonging to subgroups a-e, respectively. Class II contained thirteen *NcLBD* members, which could be further divided into two subgroups: Class IIa with six members and Class IIb with seven members (Figure 1).

Furthermore, multiple sequence alignment of the conserved LOB domains in 65 *NcLBDs* revealed that all LBDs possessed an N-terminal LOB region comprising approximately 100 amino acids (Supplementary Figure S1), consistent with findings in LBDs from sweet potato [25] and *Panax ginseng* [8]. The LOB domain is mainly composed of three conserved sequences: the zinc finger-like structure (CX2CX6CX3C), the GAS region, and the leucine-zipper-like structure (LX6LX3LX6L). Most members in Class I contained all three conserved sequences except *NcLBD16* and *NcLBD35*, which lacked the GAS region. The Class II subfamily contained the zinc finger-like structure and GAS region but lacked the leucine-zipper-like structure (Supplementary Figure S1).

### 2.2. Conserved Motif and Gene Structure Analysis of NcLBDs

**Figure 2:**
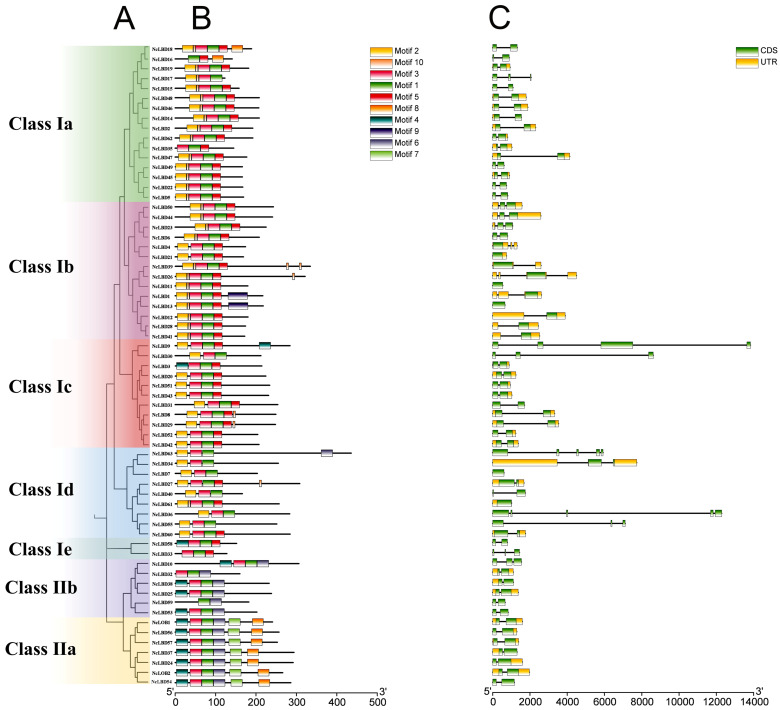
Conserved motifs and gene structure of *NcLBD* genes. (**A**) Phylogenetic tree of NcLBD protein. The phylogenetic tree was constructed based on the 65 NcLBD protein sequences using maximum likelihood (ML) method. (**B**) Conserved motif composition in NcLBD proteins. Ten motifs (motif 1~motif 10) are represented with different colored boxes. The scale bar indicated 100 amino acids. (**C**) Exon–intron structure of *NcLBD* genes. The introns are displayed in black lines, and the coding sequence and untranslated region (UTR) are shown in green and yellow boxes, respectively.

A structural analysis of the *NcLBD* genes and their corresponding proteins demonstrated unique motif compositions and gene architectures. An unrooted phylogenetic tree was constructed, showing the 65 *NcLBDs* were classified into two categories and seven subfamilies (Figure 2A). Ten conserved motifs of the NcLBD proteins were predicted using the MEME server (Figure 2B). Motif 1 and motif 3 were prevalent across all members of the *NcLBD* family, suggesting their significant role in the biological function of *NcLBDs*. In addition, the types and distributions of motifs were highly similar among various NcLBD proteins within the same clade. For instance, motifs 2 and 5 were consistently organized in Class Ia–Id, indicating a significant level of sequence similarity among the members within these clades. However, motif 6 was exclusively identified in Class II, while motif 9 was present only in *NcLBD1* and *NcLBD13*. This suggested that these motifs might play a role in the functional specificity of these *NcLBD* transcription factors.

Additionally, we analyzed the exon–intron structures of the *NcLBD* genes. As shown in Figure 2C, most of the *NcLBD* genes (69.2%) contained only one intron, including 33 genes in Class I and all members in Class II except *NcLBD10*. *NcLBD* genes within the same clade exhibited the similar exon–intron structure; two exons were present in Class II, except for *NcLBD10*, which contained three exons. However, there were also certain *NcLBD* genes that had significant structural differences from their clade counterparts. For instance, *NcLBD9* and *NcLBD30* in Class Ic had significantly more exons compared to other genes in the same clade. Moreover, large introns were also found in several *NcLBDs*, including *NcLBD9*, *NcLBD30*, *NcLBD36*, *NcLBD47*, and *NcLBD55* (Figure 2C).

### 2.3. Chromosome Localization and Collinearity Analysis of NcLBDs

**Figure 3:**
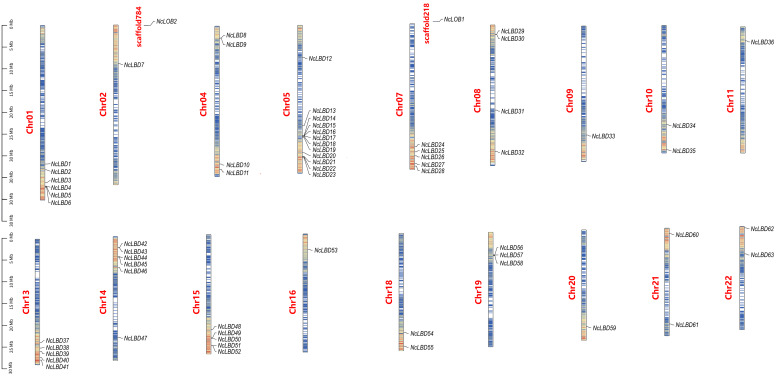
Mapping of *NcLBD* genes on the genome of *N. cadamba.* The scale bar on the left indicates 5 Mb. Gene densities are marked using color scheme, with red representing high density and blue representing low density. Chr indicates chromosome number.

The 65 *NcLBDs* from *N. cadamba* were unevenly distributed across 18 chromosomes and two unmapped scaffolds (Figure 3). Specifically, chromosome 5 harbored the highest number of genes with 12 *NcLBDs*, whereas chromosomes 2, 9, 16, and 20 each contained only one *NcLBD* gene. In addition, chromosomes 1 and 14 each possessed six *NcLBD* genes, while chromosomes 7, 13, and 15 each had five *NcLBD* genes. *NcLOB1* and *NcLOB2* were located on unmapped scaffold-784 and scaffold-218, respectively. Six pairs of *NcLBD* genes underwent tandem duplication events (*NcLBD8*/*NcLBD9*, *NcLBD16*/*NcLBD17*, *NcLBD29*/*NcLBD30*, *NcLBD42*/*NcLBD43*, *NcLBD51*/*NcLBD52*, *NcLBD56*/*NcLBD57*), located on chromosomes 4, 5, 8, 14, 15, and 19, respectively. All these tandem duplicated genes belonged to the same subfamily, exhibiting similar gene structures and conserved motifs (Figure 3).

**Figure 4:**
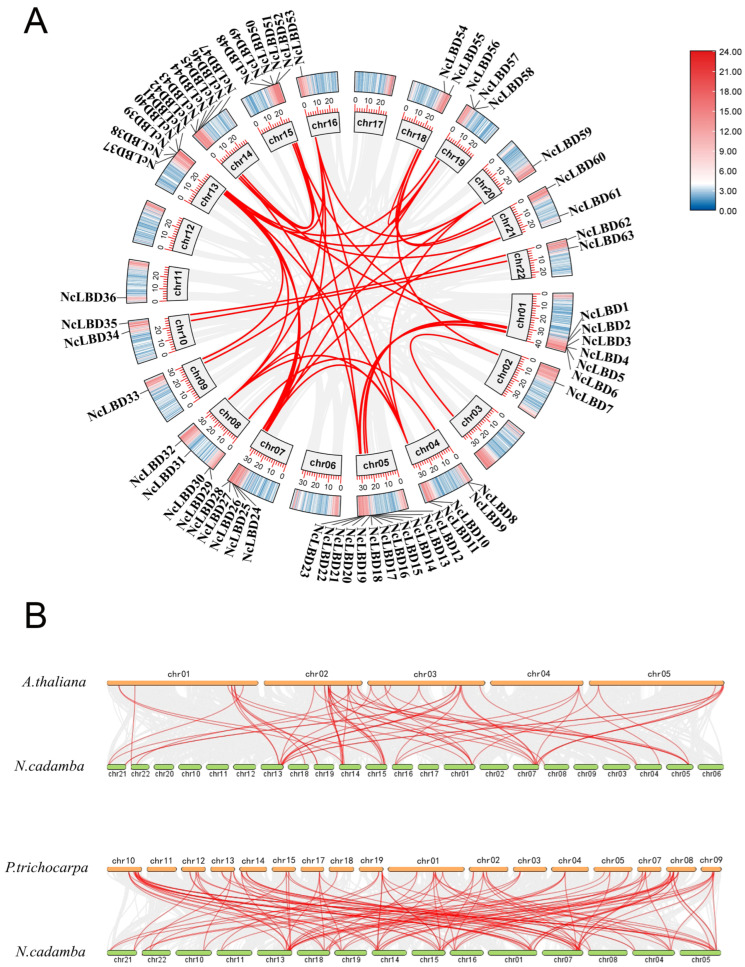
Intraspecific and interspecific collinearity of *NcLBD* genes. (**A**) Synteny of *NcLBDs* within *N. cadamba.* (**B**) Collinearity analysis of *NcLBD* genes with *A. thaliana* and *P. trichocarpa*. Gray lines in the background represent the synteny relationships in the genomes, and red lines indicate collinear LBD gene pairs.

Chromosomal or segmental duplications can lead to the expansion of gene families. Therefore, fragment duplication events within the *NcLBD* gene family were also analyzed. The result showed 58 collinear pairs among the *NcLBD* gene family, encompassing 63 *NcLBD* genes except *NcLOB1* and *NcLOB2.* The collinearity blocks were located in most chromosomes, but no linear modules were observed on chromosomes 6, 11, 12, and 17 (Figure 4A). Each pair of collinear genes was positioned on a different chromosome and was linked to fragment replication events, which likely served as the primary impetus driving the expansion of the LBD gene family.

The evolutionary distinctions among LBD genes were examined by evaluating the orthologous relationships of *NcLBD* genes in *N. cadamba* with those in the genomes of *A. thaliana* and poplar. Between *N. cadamba* and *A. thaliana*, 60 LBD orthologous gene pairs were identified, distributed across all *A. thaliana* chromosomes and 13 *N. cadamba* chromosomes (Figure 4B). Notably, the majority of collinearity blocks were localized to chromosomes 2 and 3 of *A. thaliana*. Furthermore, 116 collinear gene pairs were identified between *N. cadamba* and *P. trichocarpa*, suggesting higher collinearity between *N. cadamba* and poplar than between *N. cadamba* and *A. thaliana.* A large number of collinearity blocks were observed between chromosomes 1, 7, and 13 of *N. cadamba* and chromosomes 1, 8, and 10 of *P. trichocarpa.* Moreover, certain *NcLBD* genes, like *NcLBD37*, formed multiple orthologous gene pairs with both *P. trichocarpa* (four pairs) and *A. thaliana* (two pairs). Conversely, others displayed collinearity mainly with one species. For example, *NcLBD2* formed two pairs with *P. trichocarpa* but none with *A. thaliana*, suggesting it may have a woody plant-specific function.

### 2.4. Enrichment Analysis of Transcription Factor Binding Motif in NcLBD Promoters

**Figure 5:**
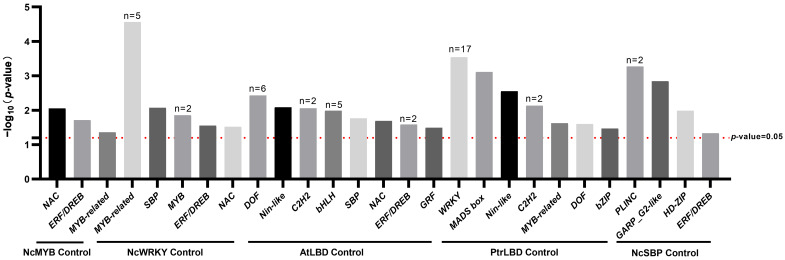
Enrichment analysis of TFBS in *NcLBD* promoters. When two or more members from a transcription factor family exhibited enrichment in the *NcLBD* promoters, the number is indicated above the bar. The red dashed line indicates *p*-value = 0.05, and the TF family was significantly enriched when *p* < 0.05.

To identify specific regulatory elements within the promoter regions of the *NcLBD* gene family, we employed JASPAR database and AME tool for motif enrichment analysis. The test group comprised 65 *NcLBD* promoter sequences, while the control group included promoter sequences from three transcription factor families in *N. cadamba* (*NcMYB*, *NcWRKY,* and *NcSBP*), as well as those from the Arabidopsis *AtLBD* gene family and the poplar *PtrLBD* gene family. The results revealed that 16 transcription factor families were significantly enriched (*p*-value < 0.05) compared to the five control groups (Figure 5). Among these, the binding motifs of the MYB-related family transcription factors, using NcWRKY as the control group, exhibited the highest level of enrichment (*p* < 0.0001) in the *NcLBD* promoters. Furthermore, when poplar *PtrLBDs* promoters were served as the control group, 17 WRKY family transcription factor binding motifs exhibited significant enrichment in *NcLBD* promoters, with *PtrWRKY45* showing the most pronounced enrichment (*p* < 0.001). Notably, when *AtLBDs* served as the control group, binding motifs from eight transcription factor families showed significant enrichment in the target gene promoter, with the most significant enrichment observed for the DOF transcription factor family (Figure 5).

### 2.5. NcLBDs Homologous Protein Interaction Network

**Figure 6:**
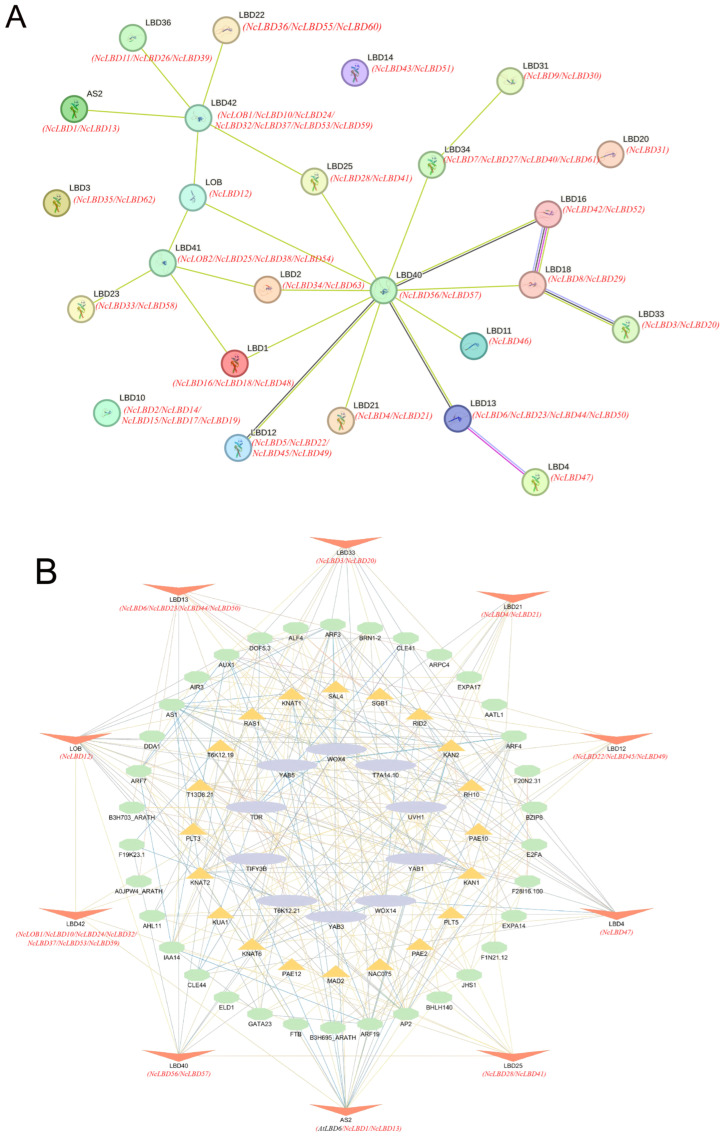
Homologous Protein Interaction Network of NcLBDs. (**A**) Interaction networks of 25 AtLBD proteins homologous to NcLBDs in *A. thaliana*. (**B**) Interaction networks of top ten AtLBD proteins highly homologous to NcLBDs in *A. thaliana*. Homologous proteins in *A. thaliana* are depicted in red; interacting proteins are shown in three other colors. Yellow lines indicate strong interactions, while blue lines denote weaker interactions.

Through homology with *Arabidopsis* AtLBDs, we utilized the STRING database to predict potential interactions among NcLBD proteins (Figure 6A). The NcLBD protein interaction network comprised 22 nodes, each interacting with others. Among them, AtLBD40 protein (orthologous with NcLBD56/NcLBD57) was predicted as a primary central node, radiating seven connections to other genes, indicating its potential core regulatory role. Furthermore, to elucidate the potential biological functions and regulatory networks of NcLBDs, ten AtLBD proteins highly homologous to NcLBDs in *A. thaliana* were employed for protein interaction network prediction (Figure 6B, homology data presented in Table S4). The result indicated that all ten AtLBD proteins were capable of interacting with over five different proteins, with AtLOB (orthologous to NcLBD12) exhibiting the highest interaction count. In addition, AtAS2 (orthologous to NcLBD1/NcLBD13), AtLBD40 (orthologous to AtNcLBD56/NcLBD57), AtLBD13 (orthologous to NcLBD6/NcLBD23/NcLBD50), AtLBD33 (orthologous to NcLBD3/NcLBD20), and AtLBD4 (orthologous to NcLBD47) also had more than ten interacting proteins, indicating their high connection to other proteins. The interacting proteins associated with hormone regulation were most presented, such as the key transcription factors ARF and PLT involved in the auxin signaling pathway.

### 2.6. Tissue-Specific Expression Patterns of NcLBDs

**Figure 7:**
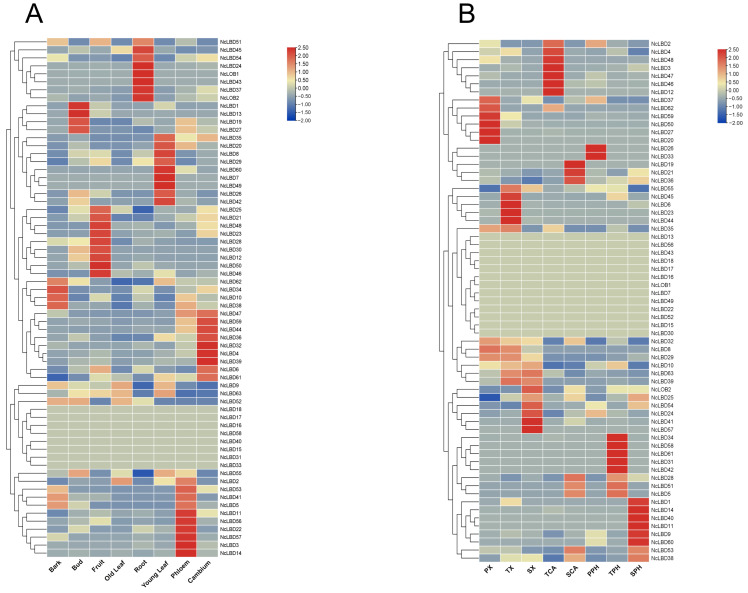
Heatmap of the expression level of *NcLBDs* in different tissues and vascular cells at three developmental stages. (**A**) Expression of *NcLBD* genes in eight different tissues from bark, bud, fruit, old leaf, young leaf, root, phloem, and cambium. (**B**) *NcLBDs* transcript levels in cambium, phloem, and xylem cells at different developmental stages including primary and secondary growth and transition stage from primary to secondary growth. PX, TX, and SX indicate the xylem cells at primary, secondary, and transitional growth stage, respectively. TCA and SCA represent the cambium cells at the transitional stage and the secondary growth stage, respectively; PPH, SPH, and TPH indicate phloem cells at the primary, secondary, and transitional growth stage, respectively. Transformed log2(FPKM + 1) values from RNA-seq data were used for heatmaps generation. Cluster analysis was analyzed by row, with the color from red to blue corresponding to high to low expression.

According to the previous public RNA-seq data [26], the expression patterns of *NcLBD* genes were investigated across eight different tissues, including buds, bark, young leaves, old leaves, roots, fruits, cambium, and phloem (Figure 7A). The results showed that the majority of *NcLBD* genes exhibited expression across a variety of tissues. Notably, *NcLBD1/13/19/27* exhibited high expression levels in buds, whereas *NcLBD10/34/38* demonstrated the highest expression in bark. *NcLBD12/21/23/25/28/30/46/48/50* was predominantly expressed in fruit, but *NcLOB1/2* and *NcLBD24/37/43/45/51/54* showed elevated expression in root, while *NcLBD7/8/20/26/29/35/42/49/60* was specifically expressed in young leaves. *NcLBD3/11/14/22/56/57* and *NcLBD4/32/36/39/44/47/49* reached their highest expression levels in phloem and cambium, respectively (Figure 7A).

In addition, the expression profiles of *NcLBDs* in various developmental vascular tissues were also explored. The analysis revealed unique spatial-temporal expression patterns of *NcLBD* genes during vascular development in *N. cadamba* (Figure 7B). Majority of the *NcLBDs* exhibited different expression levels during diverse phases of vascular development, suggesting that these *NcLBD* genes played distinct roles during wood formation. In the xylem, *NcLBD20/27/3750/59/62* exhibited elevated expression at the primary growth stage. During the transition phase, *NcLBD6/23/44/45/55* attained peak expression levels, while *NcLOB2/NcLBD24/41/54/57* demonstrated the highest expression at the secondary growth stage. Furthermore, *NcLBD6/33* had high expression during the primary growth stage of phloem development, whereas *NcLBD31/34/42/58/61* were specifically highly expressed in phloem during the transition stage, and the expression level of *NcLBD1/9/11/1440/60* was the highest in phloem at the secondary growth stage. In cambium, *NcLBD2/3/4/12/46/47/48* showed high expression in the transition stage of cambium, while *NcLBD19/1/36* were predominantly expressed in the stage of secondary growth. These distinct expression patterns of *NcLBD* genes strongly indicated that they functionally specialized in modulating specific stages of vascular tissue development and the associated differentiation processes in *N. cadamba.*

### 2.7. Expression Analysis of NcLBDs in Response to Hormones and Abiotic Stress Treatment

**Figure 8:**
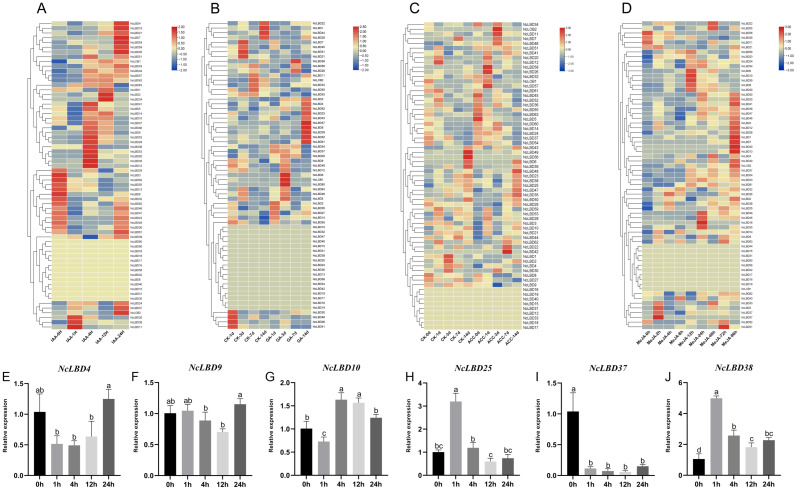
Expression pattern of *NcLBDs* at different times under various hormone treatments. (**A**–**D**) Expression levels of *NcLBDs* under IAA (auxin (indole acetic acid, (**A**)), GA (gibberellic acid, (**B**)), ACC (1-aminocyclopropane-1-carboxylic acid, precursor of ethylene (**C**)), and MeJA (methyl jasmonate, (**D**)). Heatmaps were generated based on the log2(FPKM + 1) values derived from the RNA-seq data. Genes were clustered by row, and the color bar denotes expression levels, with red and blue corresponding to high and low expression, respectively. d: day, h: hour. (**E**–**J**) Validation of expression levels of six selected *NcLBD* genes by qRT-PCR at different time points under IAA treatment. *NcUPL* was used as the reference gene, and the transcription levels at pre-treatment (0 h) were normalized to 1. Data were presented as mean ± SD (standard deviation) of three biological replicates. Significant differences among time points (ANOVA, *p* < 0.05) are indicated by different letters.

To investigate the response characteristics of *NcLBD* genes to different hormones, we analyzed the expression patterns of all identified *NcLBDs* using RNA sequencing data of the hormone treatments, including IAA, gibberellic acid (GA), 1-aminocyclopropane-1-carboxylic acid, a precursor of ethylene (ACC), and methyl jasmonate (MeJA). The results showed that under IAA treatment, *NcLBD11/25/38* exhibited induction and peak expression at 1 h, while *NcLBD1/2/34* showed induction and peak expression at 12 h. *NcLBD5/9/12/20/28/35/36/41/46/52/53* and *NcLBD4/7/19/21/24/37/43/45/54/59/NcLOB2* exhibited significant upregulation at 4 h and 24 h, respectively (Figure 8A). Among the 47 *NcLBDs* responsive to GA treatment, most exhibited an overall “initial upregulation followed by slight decline” pattern in response to GA, with 3 d being the most pronounced response time point, followed by 14 days (Figure 8B). Additionally, compared to the control group, *NcLBD26/57/58* showed significant upregulation after 1 d of ACC treatment. Notably, *NcLBD11/46* and *NcLOB2* exhibited the most pronounced upregulation trend at 3 d, while *NcLBD22/42* reached peak expression levels at 7 d (Figure 8C). For MeJA treatment, *NcLBD5/27/33/57* were upregulated as early as 2 h post-treatment. Expression of *NcLBD6/8/10/29/39/53/59* peaked at 12 h, while *NcLBD18/23/35/48/49* reached maximum expression at 24 h. *NcLBD52/51/63* and *NcLBD1/3/7/12/13/28/30/40* were significantly upregulated at 72 and 96 h post-treatment, respectively (Figure 8D). Conversely, expression levels of *NcLBD3/8/13/29/32/50/55* gradually declined with prolonged treatment duration. These results indicated that *NcLBD* genes exhibit distinct response patterns to ACC, MeJA, and IAA, suggesting that *NcLBDs* may possess unique functions in different hormone response pathways. To validate expression levels of *NcLBD* family genes under IAA treatment, *NcLBD4*, *NcLBD9*, *NcLBD10*, *NcLBD25*, *NcLBD37*, and *NcLBD38* were selected for qRT-PCR analysis (Figure 8E–J). The results revealed that the post-treatment results for *NcLBD4*, *NcLBD10*, *NcLBD25*, and *NcLBD38* were consistent with RNA sequencing data. NcLBD25/38 exhibited a pattern of initial increase followed by decrease, while *NcLBD10* showed a trend of initial decrease, subsequent increase, and then decrease. *NcLBD4* reached peak expression at 24 h, suggesting potential involvement in IAA signal transduction-related cascades. *NcLBD37* exhibited a significant decrease in expression patterns after IAA treatment.

**Figure 9:**
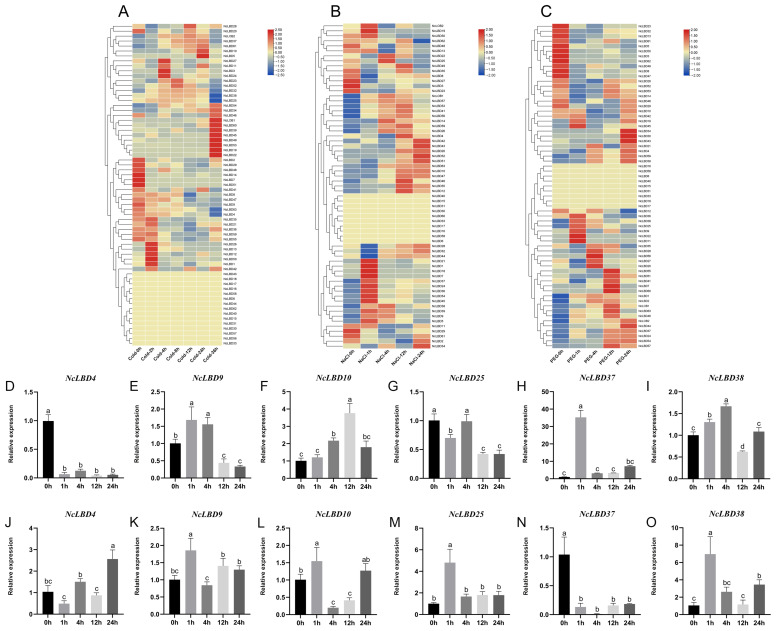
Expression profiles of *NcLBDs* in response to different abiotic stresses. (**A**–**C**) Expression pattern of *NcLBDs* in cold (**A**), salt (**B**), and drought (**C**) from RNA-seq data. Gene expression heatmaps were constructed from log2(FPKM + 1) values, with row-wise gene clustering applied. Expression levels are represented by a color bar where red indicates high expression and blue indicates low expression. (**D**–**O**) qRT-PCR analysis of six selected *NcLBD* genes after NaCl (**D**–**I**) and PEG (**J**–**O**) treatments. *NcUPL* served as the reference gene, with pre-treatment (0 h) transcription level normalized to 1 for relative quantification across other treatment groups. Error bars indicate the SD of three biological replicates. Groups denoted by different letters suggest significant differences (ANOVA, *p* < 0.05). h: hour.

To elucidate the response mechanisms of *NcLBD* genes to abiotic stresses, *NcLBDs* expression patterns were analyzed based on RNA-seq data under various stress treatments, including low temperature (4 °C), salt stress (NaCl), and drought (PEG stimulation) (Figure 9A–C). Under low-temperature stress, the expression levels of *NcLBD4/7/8/9/14/47/51/63* gradually decreased with prolonged treatment duration. In contrast, *NcLBD1/12/13/26/50* reached peak transcriptional levels at 2 h post-treatment, followed by a decline. Notably, *NcLOB1* and *NcLBD19/22/34/39/4/46/499/53/54/60* exhibited delayed responses, peaking at 24 h post-treatment, indicating a more gradual adaptation to cold stress. Under salt stress conditions, expression levels of *NcLBD3/8/23/27/29/48/61* were significantly downregulated. Conversely, *NcLOB2* and *NcLBD1/5/7/18/19/21/24/36/37/45/50/54* exhibited an initial increase at 1 h post-treatment, followed by a gradual decline. *NcLBD4/12/41/46/49/53/55/56/59* exhibited peak expression at 4 h post-treatment, while *NcLBD28/32/34/41/43/51/52* showed elevated expression only at 24 h, with lower levels at other time points. For 10% polyethylene glycol (PEG) 6000 solution treatment, expression levels of *NcLBD3/5/8/13/23/32/47/49/50/61/62* gradually decreased over time. Conversely, *NcLBD9/11/22/25/36/38/45* peaked at 1 h after treatment before declining; *NcLBD20/27/28/56* peaked at 4 h post-treatment. Concurrently, *NcLOB1* and *NcLBD7/41/46/51/55/57/60/63,* as well as *NcLOB2* and *NcLBD30/34/43/44,* exhibited maximum expression levels at 12 and 24 h post-treatment, respectively. Collectively, these findings revealed the diverse dynamic expression patterns of *NcLBD* genes in response to different abiotic stresses, suggesting they may play crucial roles in distinct stress response pathways.

To validate the expression patterns of *NcLBDs* under abiotic stress, qRT-PCR was employed to detect the expression levels of six *NcLBDs* (*NcLBD4*, *NcLBD9*, *NcLBD10*, *NcLBD25*, *NcLBD37*, and *NcLBD38*) under salt (Figure 9D–I) and drought (Figure 9J–O) stress. The results demonstrated that most *NcLBDs* showed similar expression patterns with transcriptomic data, providing crucial reference for future functional investigations of these genes. However, discrepancies were observed in certain genes (such as *NcLBD4* and *NcLBD37*) between qRT-PCR and transcriptome sequencing data. This inconsistency may stem from differences in sensitivity and quantitative principles between the two techniques, or alternatively, from alterations in RNA composition induced by the stress treatment.

## 3. Discussion

The LBD family plays a crucial role in regulating tissue development and mediating responses to environmental stimuli in plants. Owing to the rapid advancements in genome sequencing technology, the identification of LBD family has been extensively documented in various plant species. For instance, there were 43 members in *A. thaliana* [3], 36 members in rice [22], 44 members in maize [27], 43 members in potato [28], and 47 members in *Pinus massoniana* [23]. In our study, a total of 65 LBD genes were identified, indicating the *NcLBD* family has experienced significant gene expansion events. The *NcLBDs* were classified into two major groups: Class I (52 genes, 80%) and Class II (13 genes, 20%) (Figure 1). Similarly, previous studies have shown 86% of *AtLBD* genes in *A. thaliana*, 82% of *GmLBD* genes in soybeans, and 84% of *ZmLBD* genes in maize belong to Class I [3,16,26]. This pattern may be associated with the evolutionary history of the gene family, functional diversification, and adaptations specific to each species. Both segmental and tandem duplications drive the expansion of gene families and foster functional innovation [29]. This mechanism is essential for genome evolution, adapting to environmental changes, and the emergence of new species. Based on the intragenomic synteny analysis of *N. cadamba*, 58 pairs of *NcLBD* genes and 6 tandem duplicate pairs were found to have syntenic relationships. These duplication patterns contribute to functional redundancy and evolutionary diversification of gene family members (Figure 4).

The structural characteristics of *NcLBD* genes further support their functional divergence. The *NcLBD* genes contained between one and five exons, probably due to complex evolutionary events involving the insertion and deletion of introns and exons within *NcLBDs*, which may have driven functional specialization in *NcLBD* genes. Nevertheless, most genes had two exons, indicating that *NcLBDs* generally maintain a fairly conserved structure. Motifs typically denote short sequences that play key roles in biological processes. The exclusive presence of motifs 2 and 5 in Class I, motifs 4 and 6 solely in Class II, and motif 9 restricted to *NcLBD1* and *NcLBD13* suggests that these motifs may possess unique biological functions that remain to be elucidated (Figure 2). Based on the phylogenetic tree, 65 *NcLBDs* were categorized into two distinct subfamilies, resembling the phylogenetic structure observed in *AtLBDs* [3]. Class I members featured complete functional modules within their conserved domains, which typically contained an intact leucine zipper. In contrast, Class II members lacked this leucine zipper domain (Figure S1). This structural divergence paralleled the difference in subfamily-specific motif differences previously mentioned, potentially leading to distinct regulatory functions.

The LBD family plays a key role in regulating numerous biological and physiological processes in plants. To better understand the potential roles of *NcLBD* genes in the growth and development of *N. cadamba*, the expression levels of 65 *NcLBD* genes were analyzed across various tissues and different vascular cell types using transcriptome data (Figure 7). The results indicated that most LBD genes were specifically expressed in different tissues we tested, except *NcLBD15/16/17/18/31/33/40/58*. Among them, several *NcLBD* genes demonstrated high expression in fruit, root, and young leaf. Accumulating evidence indicated that LBD genes were involved in the regulation of the development of multiple organs. As shown in transgenic *Arabidopsis*, overexpression of *CsLBD37* which derived from *Camellia sinensis*, induced dwarfism, premature flowering, and reduced seed yield in *Arabidopsis* plants. Furthermore, *CsLBD37* exhibits higher expression levels in tea plant roots compared to leaves and stems, indicating its involvement in lateral root formation and signal regulation [30]. Overexpression of *PgLBD18* which derived from ginseng markedly increased the number of lateral roots and root length in transgenic *Arabidopsis*, indirectly suggesting involvement of *PgLBD18* in regulating ginseng root development [8]. *AtLBD16*, *AtLBD18*, and *AtLBD29* were key members in the formation of lateral roots and can be activated by auxin response factors *ARF7* and *ARF19*, thereby regulating the formation of lateral roots [2]. Due to the significance of wood formation in perennial trees, we focused our analysis on the expression patterns found in the xylem, cambium, and phloem across various developmental stages. Some *NcLBD* genes have high expression levels, with high overlapping, such as *NcLBD29* and *NcLBD32* displayed high expression in all the xylem cells at three various stages (Figure 7B), indicating its significant role in regulating vascular cell development. In *P. trichocarpa*, the LBD genes play crucial roles in phloem and xylem development, which have been previously reported. For example, *PtaLBD1* was highly expressed in the phloem and cambial zone. Its suppression led to a significant reduction in diameter growth and impaired phloem development [31]. Overexpression of *PagLBD21* reduced xylem width, mediated by downregulation of the genes associated with xylem development and secondary cell wall biosynthesis. Likewise, overexpression of *PagLBD4* suppressed secondary xylem differentiation and secondary cell wall deposition, whereas its knockout notably promoted both processes [14]. Additional research is required to explore the precise roles of *NcLBD29* and *NcLBD32* in the wood formation of *N. cadamba*.

Enrichment analysis of transcription factor binding motifs within the *NcLBD* promoters revealed that, in comparison to the LBD promoters of poplar and *Arabidopsis*, there was a notable enrichment of binding motifs associated with the DOF transcription factor family within the *NcLBD* gene promoters region. This finding implies the presence of conserved binding sites for the DOF transcription factor family in the *NcLBD* gene promoters (Figure 5). It has been reported that Arabidopsis DOF TFs were involved in drought stress response. For example, in *A. thaliana*, DOF4.6-XND1 can collaboratively regulate root hydraulic conductivity and drought response [32]. Specifically, we observed that *NcLBD21*, *43, 44, 45*, and *46* were all upregulated under PEG-induced drought stress and were predicted to interact with members of the DOF TF family (Figure 5 and Figure 8). This consistency between *cis*-regulatory element prediction and expression patterns suggests that these DOFs likely participate in the drought stress response by regulating the transcription of the corresponding *NcLBD* genes. Previous reports have shown that LBD TFs play crucial roles as regulators connecting hormone signaling with responses to environmental stresses. For example, salt signaling activates an auxin-independent pathway mediated by *AtLBD16* to regulate root development in *A. thaliana* [33], and *ZmLBD5* negatively regulates maize drought tolerance by impairing ABA and GA synthesis [34]. Similarly, *SlLBD40* participated in JA signal transduction during drought resistance in tomato [18]. The promoter of *ZjLBD11* was rich in JA response elements, and its expression was significantly downregulated after 48h of drought stress, indicating that *ZjLBD11* may also act as a negative regulator of drought resistance in sour jujube [4]. In our study, certain *NcLBD* genes were activated by both hormonal signals and abiotic stressors. Specifically, *NcLBD12* and *NcLBD41* were responsive to IAA and MeJA, as well as to drought and low-temperature stress. The expression of *NcLBD34* and *NcLBD43* was elevated in response to MeJA, IAA, NaCl, and PEG treatments (Figure 8 and Figure 9). In *E. grandis*, *EgLBD22*, *EgLBD29*, and *EgLBD37* exhibited responsiveness to treatments with IAA and GA [12]. These findings suggest that the LBD gene family could be important in the interaction between different plant hormones and environmental stress factors. Additionally, enrichment analysis of transcription factor binding motifs within the *NcLBD* promoters of *N. cadamba* and the potential TFs were identified (Figure 5). Among the potential TFs that may interact with *NcLBD* promoters, the WRKY family harbored the highest number of binding sites.

Numerous studies have demonstrated that members of the LBD protein family form protein interaction networks with other transcription factors, playing a pivotal role in the molecular regulation of plant growth and development. For instance, transcription factors *MYB2* and *MYB108* regulated lateral root development in *A. thaliana*, by interacting with *LBD29* [35]. *PpLBD16* formed a molecular network governing lateral root development in peach trees by binding to its downstream target genes [36]. *LBD11* controlled meristem cell proliferation in *A. thaliana*, by regulating ROS metabolism genes and modulating ROS production [13]. Furthermore, studies have proposed a WOX-ARF-LBD module that modulates plant dwarfism by influencing lateral root development in apple [37], while they also revealed a feedback regulatory pathway involving the WOX11-JMJ706-LBD16 module governing crown root development in rice [9]. Wood formation constitutes a highly complex process, involving an intricate network of pathways governing secondary growth. *Neolamarckia cadamba* is renowned for its rapid growth characteristics. Although LBD transcription factors have been studied across multiple plant species, no research has yet reported on their potential involvement in the secondary growth and development of *N. cadamba.* In our study, we predicted potential interactions among NcLBD proteins based on protein homology between *N. cadamba* and *A. thaliana*. This revealed key transcription factors associated with stress, hormone regulation, and plant growth and development (Figure 6). Within the model plant *A. thaliana*, members of the LBD protein family constitute the most extensively studied class of LBD transcription factors. *AtLBD4* and *AtLBD6* demonstrated their role in mediating hormone signaling to regulate root and stem development in *A. thaliana* [38,39]. *AtLOB* and *AtLBD33* not only interact with multiple functional proteins but may also be involved in Transmembrane Kinase (TMK)-mediated signaling cascades, participating in critical biological processes such as cell elongation, lateral root formation, and stress adaptation [40,41,42,43,44,45]. It is hypothesized that their homologues, *NcLBDs* in *N. cadamba*, possess similar functions. For instance, we observed that the genes *NcLBD37* (homologous with *AtLBD42*), *NcLBD3* (homologous with *AtLBD33*), and *NcLBD47* (homologous with *AtLBD4)* were highly expressed in the xylem and cambium (Figure 7). These three genes were also found to be induced by multiple abiotic stresses, including drought, salt, and low temperature (Figure 9). Therefore, we speculate that *NcLBD3*, *NcLBD37*, and *NcLBD47* may exert crucial regulatory functions in vascular development and the drought, cold, and salt tolerance. In summary, both the differential expression of *NcLBD* genes under diverse hormonal and abiotic stress conditions and their predicted intricate interaction networks provide substantial insights into explaining the rapid growth of *N. cadamba*. These findings offer valuable perspectives on the molecular mechanisms governing plant adaptation to stress and the regulation of vascular development.

## 4. Materials and Methods

### 4.1. Identification of NcLBD Genes in N. cadamba

The members of the LBD gene family in *N. cadamba* were identified using two methods. The amino acid sequences of 43 LBD genes in *A. thaliana* were obtained from the *Arabidopsis* Information Resource (TAIR, http://www.Arabidopsis.org/, accessed on 15 October 2024). A BLASTP search (E < 1 × 10^−5^) was conducted against the genome of *N. cadamba* using the *Arabidopsis* LBD genes as queries. Meanwhile, LOB_DNA-binding domain (PF03195) was searched to be identified by the simple HMM Search program from TBtools (version 1.0) [46]. The potential LBD proteins were cross-verified using SMART (https://smart.embl.de/, accessed on 20 October 2024) and Conserved Domain Database (CDD) in NCBI (https://www.ncbi.nlm.nih.gov/cdd/, accessed on 20 October 2024). Ultimately, 65 LBD proteins were successfully identified in *N. cadamba*. ExPASy (https://web.expasy.org/protparam/, accessed on 20 October 2024) online tools were employed to analyze the physicochemical properties of *NcLBDs* proteins, while their subcellular localization was predicted using the Plant-mPLoc website (http://www.csbio.sjtu.edu.cn/bioinf/plant-multi/, accessed on 20 October 2024) (listed in Supplementary Table S1). The genomic sequence and annotation data for *N. cadamba* used in this study are available in the NCBI BioProject database under accession number PRJNA650253.

### 4.2. Multiple Sequence Alignment and Phylogenetic Analysis of LBDs

The LOB domain of the NcLBD protein underwent multiple sequence alignment via ClustalW (https://www.genome.jp/tools-bin/clustalw, accessed on 4 November 2024) to assess its conservation. A phylogenetic tree was constructed using MEGA-X employing the maximum likelihood (ML) method followed by bootstrap analysis with 1000 replicates. The visualization and refinement of the phylogenetic tree were carried out using the Interactive Tree of Life (iTOL) (https://itol.embl.de/, accessed on 4 November 2024). According to phylogenetic relationships, the NcLBD proteins were classified into specific classes and subclasses, following the classification methods established for AtLBD [3]. The accession numbers for AtLBD proteins are provided in Supplementary Table S2.

### 4.3. Gene Structures, Conserved Motifs, and Domains Analysis

The exon–intron structures of *NcLBD* genes were analyzed and visualized in TBtools by aligning their coding sequences (CDS) with the corresponding genomic DNA sequences. Multiple Expectation Maximization for Motif Elicitation (MEME) version 5.5.7 (https://meme-suite.org/meme/index.html, accessed on 9 November 2024) was used to identify the conserved motifs of NcLBD proteins, with the number of maximum motifs set to 10. The conserved domains of NcLBD proteins were searched using the NCBI’s Conserved Domain Database (CDD) (https://www.ncbi.nlm.nih.gov/Structure/cdd/wrpsb.cgi, accessed on 9 November 2024). Visualization of the gene structure, conserved motifs, and domains were performed using TBtools (version 1.0) [46].

### 4.4. Chromosome Localization and Duplication Events of NcLBDs

The location of *NcLBDs* on the chromosome was examined and mapped using the plug-in program of Gene Location Visualize from GTF/GFF in TBtools software. To understand the duplication patterns of *NcLBDs* genes, the One Step MCScanX program from TBtools was employed to analyze the synteny between *N. cadamba* and itself, as well as between *N. cadamba* and *A. thaliana*/*P. trichocarpa*, using default parameters (E-value cut-off < 1 × 10^−10^ and Number of BlastHits with 5). Visualization of chromosome localization and synteny results were obtained using Advanced Circos within TBtools. To assess the selective pressure on *NcLBDs*, the evolutionary pressure was evaluated using the synaptonemal (Ka)/synaptonemal (Ks) ratio (Ka/Ks serves as an indicator of selective pressure). The values were calculated using the Simple KaKs Calculator (NG) within TBtools (listed in the Supplementary Table S3).

### 4.5. Motif Enrichment Analysis in NcLBD Promoters

Transcription factor binding site (TFBS) motif enrichment analysis was performed utilizing the JASPAR database (https://jaspar.elixir.no/) and the AME tool from MEME suite (https://meme-suite.org/meme/tools/ame, accessed on 27 December 2025) functional module; TFBS motif enrichment was conducted. The analysis involved 65 promoter sequences from *NcLBD* as the test group, while the control groups included promoter sequences from three transcription factor families (*NcMYB*, *NcWRKY*, *NcSBP*) derived from *N. cadamba*, as well as those from the *AtLBD* gene family of *A. thaliana* and the poplar *PtrLBD* gene family. The enrichment analysis was performed on the 2 kb upstream region of the transcription start sites for genes in both the test and control groups. Parameter settings were as follows: --verbose 1 --scoring avg --method fisher --hit-lo-fraction 0.25 --evalue -report-threshold 10.0 --control --shuffle --kmer 2. The JASPAR CORE Plant 2024 database served as the reference sequence.

### 4.6. Protein Interaction Network Analysis

Using BLASTP (https://blast.ncbi.nlm.nih.gov/Blast.cgi, accessed on 17 March 2025), all NcLBD protein sequences were compared against AtLBD protein sequences to identify direct homologues of NcLBDs (homology scores are presented in the Supplementary Table S4). Using homologous AtLBDs as references, the NcLBD protein interaction network was analyzed in the STRING (https://string-db.org/, accessed on 17 March 2025) online database (threshold score > 0.4), and further visualized and analyzed using Cytoscape software (version 3.10.3).

### 4.7. Expression Pattern of NcLBDs in Different Tissues

To analyze expression patterns of the *NcLBDs* across different tissues, transcriptome data from eight tissues including young leaves, old leaves, bud, bark, phloem, cambium, fruit, and root from 5-year-old *N. cadamba* were retrieved from our previous study [26]. In addition, three distinct types of vascular cells (cambium, phloem, and xylem) at different developmental stages (primary growth, secondary growth, and the transition from primary to secondary growth) were isolated by laser microdissection and subsequently used for RNA sequencing [26]. These RNA-seq data were downloaded from the NCBI under accession number SAMN15700859.

### 4.8. Transcriptional Profiling of NcLBDs Under Different Hormone and Stress Treatments

The treatment of *N. cadamba* seedlings with ACC has been previously described [47]. In brief, seedlings measuring 4–5 cm in height were allocated into two distinct groups: one group received ACC treatment at a concentration of 50 μmol/L (Sigma-Aldrich, St. Louis, MO, USA), while the control group was administered sterilized water. The second internode segments, counted from the apex downward, were collected for transcriptome sequencing at four time points: 6 h, 3 days (d), 7 d, and 14 d post-treatment. The RNA-seq data for ACC treatment were downloaded from Genome Sequence Archive (https://ngdc.cncb.ac.cn/gsa/, accessed on 2 June 2024) with submission number of CRA005285.

The auxin, drought, and salt stress treatments were conducted as described by Tang et al. (2025) [48]. Two-month-old seedlings of *N. cadamba* were subjected to MS liquid medium, supplied with 100 μM IAA, 10% polyethylene glycol (PEG) 6000 solution, and 100 mM NaCl, respectively. Leaf samples were collected at 1, 4, 12, and 24 h post-treatment. For MeJA treatment, the hairy roots of *N. cadamba* were cultured in the MS liquid medium with 250 μmol/L MeJA, and sampled at 2, 4, 8, 12, 24, 48, 72, and 96 h after treatment. In the cold stress experiment, three-month-old plants were placed in a growth chamber set at 4 °C. Leaf samples were collected at 2, 4, 8, 12, and 24 h intervals, with three biological replicates obtained for each time point. Total RNA was extracted from the samples and subsequently used for RNA sequencing. The expression levels of *NcLBD* genes were quantified using fragments per kilobase of exon model per million mapped reads (FPKM). The expression heat map of *NcLBDs* was generated using TBtools software. All FPKM values for each *NcLBD* gene across different tissues and treatments are presented in Supplementary Table S5.

The expression patterns of the *NcLBD* genes were validated by quantitative real-time PCR (qRT-PCR). Total RNA from leaves under different treatments was isolated and then subjected to reverse transcription using HiScript^®^ III RT SuperMix for qPCR Kit (R323, Vazyme Biotech, Nanjing, China). qRT-PCR was conducted using SYBR Green mix (Vazyme Biotech, Nanjing, China) on a LightCycler^®^ 480 instrument (Roche, Basel, Switzerland). Each sample was analyzed using three biological replicates, and each contained three technical replicates. The relative expression levels of genes were calculated by the 2^−ΔΔct^ method, and *NcUPL* (ubiquitin–protein ligase) was used as the reference gene [49]. Significance was determined by multiple comparisons using ANOVA (*p* < 0.05). The gene-specific primers used for qRT-PCR are shown in Supplementary Table S6.

## 5. Conclusions

In conclusion, we identified a total of 65 *NcLBD* genes in *N. cadamba*, which were categorized into two distinct clades based on phylogenetic analysis. We also examined the gene structures, conserved motifs, and conducted collinearity analyses. The expression profiles of *NcLBDs* across different tissues indicated their potential roles in the growth and development of *N. cadamba*. Additionally, our analysis of potential transcription factor predictions and the expression patterns of *NcLBD* genes highlighted their significant role in regulating responses to various hormones and abiotic stresses. Specifically, *NcLBD3*, *NcLBD37*, and *NcLBD47* may be associated with vascular development and cold and drought tolerance. This study provides a robust foundation for further investigation into the LBD-mediated molecular mechanisms that govern physiological developmental processes and stress responses.

## Data Availability

The original contributions presented in this study are included in the article/Supplementary Materials. Further inquiries can be directed to the corresponding author.

## Supplementary Materials

The following supporting information can be downloaded at: https://www.mdpi.com/article/10.3390/ijms27020693/s1.

## References

[1] Du Y., Zhao Q., Li W., Geng J., Li S., Yuan X., Gu Y., Zhong J., Zhang Y., Du J. (2022). Genome-wide identification of the LBD transcription factor genes in common bean (*Phaseolus vulgaris* L.) and expression analysis under different abiotic stresses. J. Plant Interact..

[2] Zhang Y., Li Z., Ma B., Hou Q., Wan X. (2020). Phylogeny and Functions of LOB Domain Proteins in Plants. Int. J. Mol. Sci..

[3] Shuai B., Reynaga-Peña C.G., Springer P.S. (2002). The *Lateral Organ Boundaries* Gene Defines a Novel, Plant-Specific Gene Family. Plant Physiol..

[4] Li M., Zhang Q., Zhou S., Wang R., Zhao Y., Geng J. (2025). Genome-wide characterization and expression analysis of LBD transcription factors in *Ziziphus jujuba* var. spinosa: Putative roles in tissue development and abiotic stress adaptation. Front. Plant Sci..

[5] Wang R., Bai T., Gao H., Cui Y., Zhou R., Wang Z., Song S., Jiao J., Wang M., Wan R. (2023). Genome-wide identification of *LBD* transcription factors in apple and the function of *MdLBD16a* in adventitious rooting and callus development. Sci. Hortic..

[6] Dang H., Yu C., Nan S., Li Y., Du S., Zhao K., Wang S. (2024). Genome-wide identification and gene expression networks of LBD transcription factors in *Populus trichocarpa*. BMC Genom..

[7] Okushima Y., Fukaki H., Onoda M., Theologis A., Tasaka M. (2007). ARF7 and ARF19 Regulate Lateral Root Formation via Direct Activation of *LBD*/*ASL* Genes in *Arabidopsis*. Plant Cell.

[8] Wang Y., Wang P., Di P., Wang Y. (2025). Genome-wide analysis of *LBD* genes in the medicinal plant *Panax ginseng* reveals the roles and molecular mechanisms of *PgLBD18* and *PgLBD49* in regulating lateral root development. Ind. Crops Prod..

[9] Geng L., Tan M., Deng Q., Wang Y., Zhang T., Hu X., Ye M., Lian X., Zhou D., Zhao Y. (2024). Transcription factors WOX11 and LBD16 function with histone demethylase JMJ706 to control crown root development in rice. Plant Cell.

[10] Kim M.J., Kim M., Lee M.R., Park S.K., Kim J. (2015). *LATERAL ORGAN BOUNDARIES DOMAIN* (*LBD*)_*10*_ interacts with *SIDECAR POLLEN*/*LBD_27_* to control pollen development in Arabidopsis. Plant J..

[11] Wang X., Zhang S., Su L., Liu X., Hao Y. (2013). A genome-wide analysis of the LBD (LATERAL ORGAN BOUNDARIES domain) gene family in *Malus domestica* with a functional characterization of *MdLBD11*. PLoS ONE.

[12] Lu Q., Shao F., Macmillan C., Wilson I.W., van der Merwe K., Hussey S.G., Myburg A.A., Dong X., Qiu D. (2018). Genomewide analysis of the lateral organ boundaries domain gene family in *Eucalyptus grandis* reveals members that differentially impact secondary growth. Plant Biotechnol. J..

[13] Dang T.V.T., Lee S., Cho H., Choi K., Hwang I. (2023). The LBD11-ROS feedback regulatory loop modulates vascular cambium proliferation and secondary growth in *Arabidopsis*. Mol. Plant.

[14] Li H., Yin S., Wang L., Xu N., Liu L. (2022). Transcription factor PagLBD21 functions as a repressor of secondary xylem development in *Populus*. For. Res..

[15] Han Z., Yang T., Guo Y., Cui W., Yao L., Li G., Wu A., Li J., Liu L. (2021). The transcription factor PagLBD3 contributes to the regulation of secondary growth in *Populus*. J. Exp. Bot..

[16] Yang H., Shi G., Du H., Wang H., Zhang Z., Hu D., Wang J., Huang F., Yu D. (2017). Genome-Wide Analysis of Soybean *LATERAL ORGAN BOUNDARIES Domain* -Containing Genes: A Functional Investigation of *GmLBD12*. Plant Genome.

[17] Guo Z., Xu H., Lei Q., Du J., Li C., Wang C., Yang Y., Yang Y., Sun X. (2020). The Arabidopsis transcription factor LBD15 mediates ABA signaling and tolerance of water-deficit stress by regulating *ABI4* expression. Plant J..

[18] Liu L., Zhang J., Xu J., Li Y., Guo L., Wang Z., Zhang X., Zhao B., Guo Y., Zhang N. (2020). CRISPR/Cas9 targeted mutagenesis of *SlLBD40*, a lateral organ boundaries domain transcription factor, enhances drought tolerance in tomato. Plant Sci..

[19] Cao H., Liu C., Liu C., Zhao Y., Xu R. (2016). Genomewide analysis of the lateral organ boundaries domain gene family in *Vitis vinifera*. J. Genet..

[20] Wei Y., Lin Z., Jin J., Zhu W., Gao J., Li J., Xie Q., Lu C., Zhu G., Yang F. (2025). Genome-wide identification and functional characterization of LBD gene family in four *Cymbidium* species (Orchidaceae) and potential regulatory role of *CsiLBD27* in floral development of *Cymbidium sinense*. BMC Genom..

[21] Xu J., Hu P., Tao Y., Song P., Gao H., Guan Y. (2021). Genome-wide identification and characterization of the *Lateral Organ Boundaries Domain* (*LBD* ) gene family in polyploid wheat and related species. PeerJ.

[22] Zhao D., Chen P., Chen Z., Zhang L., Wang Y., Xu L. (2023). Genome-wide analysis of the LBD family in rice: Gene functions, structure and evolution. Comput. Biol. Med..

[23] Zhang C., Zhu P., Zhang M., Huang Z., Hippolyte A.R., Hou Y., Lou X., Ji K. (2022). Identification, Classification and Characterization of LBD Transcription Factor Family Genes in Pinus massoniana. Int. J. Mol. Sci..

[24] Gong S., Ho W.S., Yue J., Liu L. (2023). Comparative Analysis on Adaptability of Different Ploidy *Neolamarckiacadamba* to Low Temperature Stress. J. Nat. Sci. Biol. Med..

[25] Shi L., Lin X., Tang B., Zhao R., Wang Y., Lin Y., Wu L., Zheng C., Zhu H. (2024). Genome-Wide Analysis of the Lateral Organ Boundaries Domain (LBD) Gene Family in Sweet Potato (*Ipomoea batatas*). Genes.

[26] Zhao X., Hu X., OuYang K., Yang J., Que Q., Long J., Zhang J., Zhang T., Wang X., Gao J. (2022). Chromosome-level assembly of the *Neolamarckia cadamba* genome provides insights into the evolution of cadambine biosynthesis. Plant J..

[27] Wang S., Wang Y., Zhong J., Xu W., Gong Q., Zhai L., Li G., Huang J. (2025). Genome-Wide Analysis of the Maize LBD Gene Family Reveals a Role for *ZmLBD12* in the Development of Lateral Roots. Plants.

[28] Liu H., Cao M., Chen X., Ye M., Zhao P., Nan Y., Li W., Zhang C., Kong L., Kong N. (2019). Genome-Wide Analysis of the Lateral Organ Boundaries Domain (LBD) Gene Family in *Solanum tuberosum*. Int. J. Mol. Sci..

[29] Banks D.R.S.J. (2014). Gene duplication as a driver of plant morphogenetic evolution Stefan A Rensing. Plant Biol..

[30] Teng R., Yang N., Liu C., Chen Y., Wang Y., Zhuang J. (2022). *CsLBD37*, a LBD/ASL transcription factor, affects nitrate response and flowering of tea plant. Sci. Hortic..

[31] Baldacci Cresp F., Moussawi J., Leplé J.C., Van Acker R., Kohler A., Candiracci J., Twyffels L., Spokevicius A.V., Bossinger G., Laurans F. (2015). PtaRHE 1, a *Populus tremula* × *Populus alba* RING -H2 protein of theATL family, has a regulatory role in secondary phloem fibre development. Plant J..

[32] Ding B., Liang M., Shi Y., Zhang R., Wang J., Huang Y., Yan D., Hou X., Maurel C., Tang N. (2025). The Transcription Factors DOF4.6 and XND1 Jointly Regulate Root Hydraulics and Drought Responses in *Arabidopsis*. Plant Cell.

[33] Zhang Y., Li Y., de Zeeuw T., Duijts K., Kawa D., Lamers J., Munzert K.S., Li H., Zou Y., Meyer A.J. (2024). Root branching under high salinity requires auxin-independent modulation of LATERAL ORGAN BOUNDARY DOMAIN 16 function. Plant Cell.

[34] Feng X., Xiong J., Zhang W., Guan H., Zheng D., Xiong H., Jia L., Hu Y., Zhou H., Wen Y. (2022). *ZmLBD5*, aclass-II *LBD* gene, negatively regulates drought tolerance by impairing abscisic acid synthesis. Plant J..

[35] Zhang F., Wang J., Ding T., Lin X., Hu H., Ding Z., Tian H. (2024). MYB2 and MYB108 regulate lateral root development by interacting with LBD29 in *Arabidopsis thaliana*. J. Integr. Plant Biol..

[36] Wu X., Wang Z., Du A., Gao H., Liang J., Yu W., Yu H., Fan S., Chen Q., Guo J. (2024). Transcription factor LBD16 targets cell wall modification/ion transport genes in peach lateral root formation. Plant Physiol..

[37] Lv J., Feng Y., Zhai L., Jiang L., Wu Y., Huang Y., Yu R., Wu T., Zhang X., Wang Y. (2024). *MdARF3* switches the lateral root elongation to regulate dwarfing in apple plants. Hortic. Res..

[38] Ye L., Wang X., Lyu M., Siligato R., Eswaran G., Vainio L., Blomster T., Zhang J., Mähönen A.P. (2021). Cytokinins initiate secondary growth in the *Arabidopsis* root through a set of LBD genes. Curr. Biol..

[39] Guo M., Thomas J., Collins G., Timmermans M.C.P. (2008). Direct Repression of KNOX Loci by the ASYMMETRIC LEAVES1 Complex of *Arabidopsis*. Plant Cell.

[40] Gendron J.M., Liu J., Fan M., Bai M., Wenkel S., Springer P.S., Barton M.K., Wang Z. (2012). Brassinosteroids regulate organ boundary formation in the shoot apical meristem of *Arabidopsis*. Proc. Natl. Acad. Sci. USA.

[41] Husbands A., Bell E.M., Shuai B., Smith H.M.S., Springer P.S. (2007). LATERAL ORGAN BOUNDARIES defines a new family of DNA-binding transcription factors and can interact with specific bHLH proteins. Nucleic Acids Res..

[42] Yu Q., Hu S., Du J., Yang Y., Sun X. (2020). Genome-wide identification and characterization of the lateral organ boundaries domain gene family in *Brassica rapa* var. *rapa*. Plant Divers..

[43] Zhang Y., Ma Y., Zhao D., Tang Z., Zhang T., Zhang K., Dong J., Zhang H. (2023). Genetic regulation of lateral root development. Plant Signal. Behav..

[44] Lin W., Zhou X., Tang W., Takahashi K., Pan X., Dai J., Ren H., Zhu X., Pan S., Zheng H. (2021). TMK-based cell-surface auxin signalling activates cell-wall acidification. Nature.

[45] Peng Y., Tan S. (2021). TMK: A crucial piece of the acid growth puzzle. Mol. Plant.

[46] Chen C., Chen H., Zhang Y., Thomas H.R., Frank M.H., He Y., Xia R. (2020). TBtools: An Integrative Toolkit Developed for Interactive Analyses of Big Biological Data. Mol. Plant.

[47] Yi N., Yang H., Zhang X., Pian R., Li H., Zeng W., Wu A. (2022). The physiological and transcriptomic study of secondary growth in *Neolamarckia cadamba* stimulated by the ethylene precursor ACC. Plant Physiol. Biochem..

[48] Tang L., Li K., Cai C., Wu W., Jian G., Lei Z., Peng C., Long J. (2025). Genome-Wide Identification and Expression Pattern Analysis of SBP Gene Family in *Neolamarckia cadamba*. Genes.

[49] Zhang D., Li J.J., Zhang M.J., Bao Y.T., Yang X., Xu W.Y., Quyang K.X., Chen X.Y. (2018). Selection and validation of reference genes for quantitative RT-PCR analysis in *Neolamarckia cadamba*. Chin. Bull. Bot..

