# Supplementary material for: Genome-Wide Identification and Expression Analysis of LBD Gene Family in Neolamarckia cadamba
Source: Int J Mol Sci. 2026 Jan 9;27(2):693. doi: 10.3390/ijms27020693 (PMC12841386; doi:10.3390/ijms27020693)
Supplement: Supplementary file 1 [file ijms-27-00693-s001.zip › Figure S1.pdf]

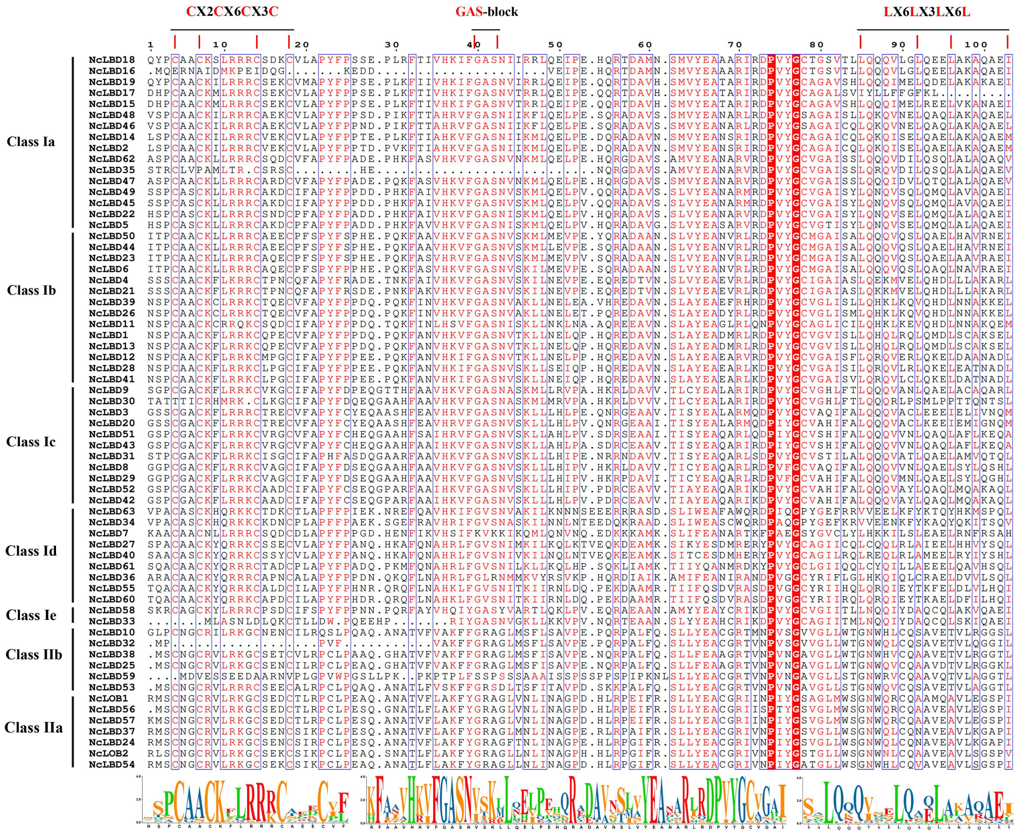

Figure S1. Alignment of LOB domains of NcLBD proteins. The CX2CX6CX3C zinc finger-like domain, the GAS-block, and the leucine zipper-like motif (LX6LX3LX6L) were presented at the top of the diagram, and the conservative structural domain identifier was generated by the WebLogo programme at the bottom.
