# Supplementary material for: Genome-Wide Identification and Expression Analysis of LBD Gene Family in Neolamarckia cadamba
Source: Int J Mol Sci. 2026 Jan 9;27(2):693. doi: 10.3390/ijms27020693 (PMC12841386; doi:10.3390/ijms27020693)
Supplement: Supplementary file 1 [file ijms-27-00693-s001.zip › Table S1.pdf]

Table S1 Physicochemical properties of LBD protein in *N. cadamba*

Sequence ID	Number of amino acids(aa)	Molecular weight (Da)	Theoretical (pl)	Instability Index	Aliphatic Index	Grand average of hydropathicity
NcLBD1	216	23506.43	8.55	50.92	74.63	-0.392
NcLBD2	191	20822.83	5.39	63.17	81.31	-0.185
NcLBD3	213	23977.03	4.34	56.86	80.61	-0.064
NcLBD4	173	19367.9	5.24	51.69	80.06	-0.323
NcLBD5	168	18530.08	5.37	55.62	80.71	-0.161
NcLBD6	207	21883.09	8.88	76.74	84.93	-0.039
NcLBD7	202	22500.34	6.95	54.31	68.17	-0.65
NcLBD8	248	25650.08	7.06	72.84	77.54	-0.058
NcLBD9	823	91941.53	5.96	47.58	70.74	-0.522
NcLBD10	305	33638.48	9.09	56.33	79.97	-0.325
NcLBD11	179	19742.75	8.48	69.05	74.19	-0.403
NcLBD12	179	19674.25	7.07	65.15	62.18	-0.478
NcLBD13	217	23778.71	8.55	51.58	71.15	-0.424
NcLBD14	207	22595.81	5.24	69.7	77.39	-0.242
NcLBD15	157	17916.6	8.87	70.35	73.31	-0.426
NcLBD16	140	16062.01	4.14	48.01	88.43	-0.441
NcLBD17	122	13895.96	8.63	67.05	71.15	-0.218
NcLBD18	188	21463.41	4.98	65.24	83.51	-0.306
NcLBD19	181	20623.45	5.07	68.22	72.21	-0.267
NcLBD20	223	24892.21	4.67	54.93	79.69	-0.102
NcLBD21	168	18883.53	6.14	59.2	76.67	-0.279
NcLBD22	166	18383.91	5.54	59.06	81.69	-0.197
NcLBD23	224	24242.02	9.05	72.3	84.96	-0.067
NcLBD24	291	31961.76	6.28	48.1	73.09	-0.584
NcLBD25	237	25928.49	7.52	51.61	79.03	-0.276
NcLBD26	320	35685.23	7.21	67.97	59.56	-0.706

Table S1 Physicochemical properties of LBD protein in *N. cadamba*

Sequence ID	Number of amino acids(aa)	Molecular weight (Da)	Theoretical (pl)	Instability Index	Aliphatic Index	Grand average of hydropathicity
NcLBD27	307	34617.78	5.58	53.27	67.36	-0.506
NcLBD28	173	19192.54	8.27	50.73	61.56	-0.551
NcLBD29	247	25492.88	7.13	64.47	76.36	-0.06
NcLBD30	211	22980.49	7.67	42.44	80	-0.02
NcLBD31	253	27297.25	4.91	57.75	75.65	-0.331
NcLBD32	159	17435.91	8.4	67.2	77.86	-0.19
NcLBD33	127	14064.94	5.23	24.7	86.85	-0.227
NcLBD34	254	27982.08	9.41	35.53	56.18	-0.738
NcLBD35	144	15937.77	6.49	64.21	62.29	-0.428
NcLBD36	494	52964.59	4.61	45.4	70.49	-0.417
NcLBD37	293	32390.21	5.66	49.8	71.95	-0.642
NcLBD38	231	25058.47	8.19	52.14	78.53	-0.281
NcLBD39	333	37434.22	7.27	63.89	60.99	-0.683
NcLBD40	165	18420.07	9.14	49.84	78.06	-0.355
NcLBD41	171	18897.17	6.19	53.77	58.83	-0.548
NcLBD42	206	22503.75	8.02	50.58	70.73	-0.177
NcLBD43	230	25414.39	5.75	47.98	61.57	-0.366
NcLBD44	240	25942.58	8.76	80.02	71.62	-0.344
NcLBD45	165	18297.01	6.7	52.62	88.12	-0.13
NcLBD46	206	22356.35	6.09	70.41	71.6	-0.215
NcLBD47	176	19425.89	8.6	58.37	62.67	-0.489
NcLBD48	207	22509.41	5.58	69.57	71.74	-0.259
NcLBD49	165	18304.02	6.08	53.54	82.18	-0.142
NcLBD50	242	25935.57	8.6	72.89	71.86	-0.239
NcLBD51	233	25946.06	5.75	48.85	63.26	-0.349
NcLBD52	203	22157.42	8.63	50.07	74.19	-0.165

Table S1 Physicochemical properties of LBD protein in *N. cadamba*

Sequence ID	Number of amino acids(aa)	Molecular weight (Da)	Theoretical (pl)	Instability Index	Aliphatic Index	Grand average of hydropathicity
NcLBD53	201	21537.35	4.95	64.52	72.84	-0.068
NcLBD54	285	30391.27	6.66	46.65	77.33	-0.36
NcLBD55	250	28382.44	5.74	52.18	91.72	-0.27
NcLBD56	256	28115.84	7.61	56.3	79	-0.404
NcLBD57	252	27986.78	6.07	52.01	83.17	-0.38
NcLBD58	151	17012.38	8.45	50.63	77.55	-0.321
NcLBD59	181	19095.49	4.62	59.18	75.97	-0.213
NcLBD60	283	32099.15	6.34	47.46	73.82	-0.548
NcLBD61	256	28980.63	6.03	63.62	72.89	-0.545
NcLBD62	191	21146.9	6.37	71.52	66.91	-0.474
NcLBD63	434	48023.19	9.31	49.44	69.86	-0.593
NcLOB1	240	26705.61	7.55	37.26	82.08	-0.309
NcLOB2	265	28311.13	8.1	45.84	82.75	-0.287
