# Supplementary material for: Genome-Wide Identification and Expression Analysis of LBD Gene Family in Neolamarckia cadamba
Source: Int J Mol Sci. 2026 Jan 9;27(2):693. doi: 10.3390/ijms27020693 (PMC12841386; doi:10.3390/ijms27020693)
Supplement: Supplementary file 1 [file ijms-27-00693-s001.zip › Table S2.pdf]

Table S2 The accession numbers of the AtLBD proteins used for ML phylogenetic tree construction

Species	Gene Name	Accession no.	Database
Arabidopsis thaliana	*AtLBD1*	AT1G07900.1	TAIR
	AtLBD2	AT1G06280.1	TAIR
	AtLBD3	AT1G16530.1	TAIR
	AtLBD4	AT1G31320.1	TAIR
	AtLBD5	AT1G36000.1	TAIR
	AtLBD6	AT1G65620.1	TAIR
	AtLBD7	AT1G72980.1	TAIR
	AtLBD8	AT2G19510.1	TAIR
	AtLBD9	AT2G19820.1	TAIR
	AtLBD10	AT2G23660.1	TAIR
	AtLBD11	AT2G28500.1	TAIR
	AtLBD12	AT2G30130.1	TAIR
	AtLBD13	AT2G30340.2	TAIR
	AtLBD14	AT2G31310.1	TAIR
	AtLBD15	AT2G40470.1	TAIR
	AtLBD16	AT2G42430.1	TAIR
	AtLBD17	AT2G42440.1	TAIR
	AtLBD18	AT2G45420.1	TAIR
	AtLBD19	AT2G45410.1	TAIR
	AtLBD20	AT3G03760.1	TAIR
	AtLBD21	AT3G11090.1	TAIR
	AtLBD22	AT3G13850.1	TAIR
	AtLBD23	AT3G26620.1	TAIR
	AtLBD24	AT3G26660.1	TAIR
	AtLBD25	AT3G27650.1	TAIR
	AtLBD26	AT3G27940.1	TAIR
	AtLBD27	AT3G47870.1	TAIR
	AtLBD28	AT3G50510.1	TAIR
	AtLBD29	AT3G58190.1	TAIR
	AtLBD30	AT4G00220.1	TAIR
	AtLBD31	AT4G00210.1	TAIR
	AtLBD32	AT4G22700.1	TAIR
	AtLBD33	AT5G06080.1	TAIR
	AtLBD34	AT5G15060.1	TAIR
	AtLBD35	AT5G35900.1	TAIR
	AtLBD36	AT5G66870.1	TAIR
	AtLBD37	AT5G67420.1	TAIR
	AtLBD38	AT3G49940.1	TAIR
	AtLBD39	AT4G37540.1	TAIR
	AtLBD40	AT1G67100.1	TAIR
	AtLBD41	AT3G02550.1	TAIR

Table S2 The accession numbers of the AtLBD proteins used for ML phylogenetic tree construction

Species	Gene Name	Accession no.	Database
Arabidopsis thaliana	*AtLBD42*	AT1G68510.1	TAIR
	AtLOB	AT5G63090.1	TAIR
