# Supplementary material for: Genome-Wide Identification and Expression Analysis of LBD Gene Family in Neolamarckia cadamba
Source: Int J Mol Sci. 2026 Jan 9;27(2):693. doi: 10.3390/ijms27020693 (PMC12841386; doi:10.3390/ijms27020693)
Supplement: Supplementary file 1 [file ijms-27-00693-s001.zip › Table S3.pdf]

Table S3 Ka/Ks ratio for collinear genes within species of the NcLBD family

Seq_1	Seq_2	Ka_Ks	pS	Note
NcLBD2	NcLBD14	0.17605394070819363	0.31847133757961815	
NcLBD3	NcLBD20	0.24757095431296028	0.3618181818181821	
NcLBD4	NcLBD21	0.1406374680214159	0.3755334281650074	
NcLBD5	NcLBD22	0.16832373580754384	0.2717311906501098	
NcLBD6	NcLBD23	0.11746072365094862	0.4328799526907155	
NcLBD1	NcLBD13	0.03827851507996966	0.3557951482479786	
NcLBD5	NcLBD45	0.1284999684495709	0.5932075471698118	
NcLBD6	NcLBD44	0.0994937555666986	0.6521008403361342	
NcLBD5	NcLBD49	0.1078470636993507	0.655849056603774	
NcLBD6	NcLBD50	0.12027026929479062	0.6119235095613046	
NcLBD10	NcLBD25	NaN	0.8055908513341811	High Sequence Divergence Value (pS>=0.75)
NcLBD10	NcLBD32	0.2059539495754741	0.3945530726256986	
NcLBD8	NcLBD29	0.13448383470052483	0.4096916299559469	
NcLBD10	NcLBD38	0.11781529349617341	0.7271073377804732	
NcLBD10	NcLBD53	0.18190872549161685	0.6910994764397912	
NcLBD10	NcLBD59	0.2864164353922664	0.6980988593155899	
NcLBD22	NcLBD45	0.07049547628877337	0.7075038284839209	
NcLBD23	NcLBD44	0.13231798919846516	0.6155106499180776	
NcLBD22	NcLBD49	0.07843930404522881	0.6873065015479882	
NcLBD23	NcLBD50	0.11053828825561135	0.6591549295774647	
NcLBD25	NcLBD32	0.10187123466991535	0.7455325232308799	
NcLBD24	NcLBD37	0.17840800938028184	0.3294892915980226	
NcLBD25	NcLBD38	0.2889513846893939	0.28571428571428564	
NcLBD26	NcLBD39	0.15566911841525746	0.4310954063604232	
NcLBD27	NcLBD40	0.35630555515707707	0.29950900163666133	

Table S3 Ka/Ks ratio for collinear genes within species of the NcLBD family

Seq_1	Seq_2	Ka_Ks	pS	Note
NcLBD28	NcLBD41	0.1905318915345266	0.25923244026068093	
NcLBD25	NcLBD53	0.2740235425750821	0.6619217081850535	
NcLBD24	NcLBD56	0.15762530872931987	0.7008928571428569	
NcLBD27	NcLBD61	0.17497682993042077	0.719017094017094	
NcLBD32	NcLBD38	0.187207444922064	0.6740905716406838	
NcLBD32	NcLBD53	0.30783088469792497	0.61764705882353	
NcLBD32	NcLBD59	0.2686570159044541	0.6915339480301765	
NcLBD33	NcLBD58	0.5276093383783342	0.35426429240862245	
NcLBD35	NcLBD62	0.24446855488944016	0.475945017182131	
NcLBD34	NcLBD63	0.3783215317435404	0.32954545454545425	
NcLBD38	NcLBD53	0.15139637471395775	0.7216676453317677	
NcLBD38	NcLBD59	NaN	0.8001297858533429	High Sequence Divergence Value (pS>=0.75)
NcLBD40	NcLBD61	NaN	0.7867338371116714	High Sequence Divergence Value (pS>=0.75)
NcLBD42	NcLBD51	NaN	0.8092369477911657	High Sequence Divergence Value (pS>=0.75)
NcLBD44	NcLBD50	0.11585765714237467	0.3316110850164396	
NcLBD45	NcLBD49	0.07712067432586092	0.27637444279346235	
NcLBD46	NcLBD48	0.08584301388506856	0.3721474546518433	
NcLBD53	NcLBD59	0.34946195401843394	0.4756504336224154	
NcLBD54	NcLBD56	0.1778204680040532	0.6971177332681971	
NcLBD55	NcLBD60	0.3215513916970255	0.4044444444444445	
