# Supplementary material for: Genome-Wide Identification and Expression Analysis of LBD Gene Family in Neolamarckia cadamba
Source: Int J Mol Sci. 2026 Jan 9;27(2):693. doi: 10.3390/ijms27020693 (PMC12841386; doi:10.3390/ijms27020693)
Supplement: Supplementary file 1 [file ijms-27-00693-s001.zip › Table S4.pdf]

Table S4 Comparison of Protein Homology Between NcLBDs and AtLBDs

suject	query	Total Score	Query Cover	E-value	idengty	Acc.len
NcLBD1	AtLBD6	253	99%	1e-90	67.44%	216
NcLBD2	AtLBD10	142	33%	9e-46	56.73%	192
NcLBD3	AtLBD33	174	61%	7e-60	72.22%	214
NcLBD4	AtLBD21	186	67%	2e-65	77.48%	174
NcLBD5	AtLBD12	221	77%	7e-79	72.48%	169
NcLBD6	AtLBD13	182	42%	1e-61	80.53%	208
NcLBD7	AtLBD34	64.7	0.52	2E-18	40.79%	203
NcLBD8	AtLBD18	231	83%	1e-80	59.36%	249
NcLBD9	AtLBD31	199	98%	2e-63	49.55%	824
NcLBD10	AtLBD42	150	45%	2e-48	61.32%	306
NcLBD11	AtLBD36	184	55%	4e-62	54.29%	180
NcLBD12	AtLOB	217	66%	8e-78	86.07%	179
NcLBD13	AtLBD6	257	99%	3e-92	65.16%	217
NcLBD14	AtLBD10	143	33%	9e-46	50.43%	208
NcLBD15	AtLBD10	139	38%	6e-45	50.43%	158
NcLBD16	AtLBD1	85.5	54%	4e-26	51.92%	141
NcLBD17	AtLBD10	125	27%	8e-40	61.45%	123
NcLBD18	AtLBD1	168	88%	6e-58	53.85%	189
NcLBD19	AtLBD10	142	39%	7e-46	50.82%	182
NcLBD20	AtLBD33	179	69%	8e-62	67.19%	224
NcLBD21	AtLBD21	191	67%	1e-67	80.18%	169
NcLBD22	AtLBD12	222	77%	2e-79	73.83%	167
NcLBD23	AtLBD13	218	56%	3e-75	64.81%	225
NcLBD24	AtLBD42	216	61%	7e-74	68.53%	292
NcLBD25	AtLBD41	170	42%	1e-56	66.36%	238
NcLBD26	AtLBD36	255	78%	6e-88	56.42%	321

Table S4 Comparison of Protein Homology Between NcLBDs and AtLBDs

suject	query	Total Score	Query Cover	E-value	idengty	Acc.len
NcLBD27	AtLBD34	84	56%	2e-24	48.10%	308
NcLBD28	AtLBD25	209	70%	6e-75	86.49%	174
NcLBD29	AtLBD18	234	79%	6e-82	63.64%	248
NcLBD30	AtLBD31	150	92%	1e-49	42.93%	212
NcLBD31	AtLBD20	249	82%	2e-87	61.60%	254
NcLBD32	AtLBD42	102	57%	4e-31	44.53%	160
NcLBD33	AtLBD23	159	96%	7e-56	64.35%	152
NcLBD34	AtLBD2	121	44%	3e-38	55.91%	255
NcLBD35	AtLBD3	87.4	33%	1e-26	69.09%	169
NcLBD36	AtLBD22	135	38%	8e-41	55.88%	495
NcLBD37	AtLBD42	215	59%	2e-74	68.53%	294
NcLBD38	AtLBD41	166	42%	6e-55	65.45%	232
NcLBD39	AtLBD36	270	78%	1e-93	57.31%	334
NcLBD40	AtLBD34	72.4	51%	5e-21	43.84%	166
NcLBD41	AtLBD25	204	70%	5e-73	85.59%	172
NcLBD42	AtLBD16	220	96%	1e-73	52.89%	207
NcLBD43	AtLBD14	187	98%	2e-64	53.30%	231
NcLBD44	AtLBD13	231	64%	2e-80	68.60%	241
NcLBD45	AtLBD12	246	98%	4e-89	70.16%	165
NcLBD46	AtLBD11	203	82%	3e-71	65.06%	207
NcLBD47	AtLBD4	252	100%	7e-92	72.32%	177
NcLBD48	AtLBD1	204	82%	1e-71	64.46%	208
NcLBD49	AtLBD12	246	98%	4e-89	70.16%	165
NcLBD50	AtLBD13	225	55%	6e-28	74.32%	243
NcLBD51	AtLBD14	173	76%	4e-59	57.53%	234
NcLBD52	AtLBD16	211	96%	3e-77	54.62%	204

Table S4 Comparison of Protein Homology Between NcLBDs and AtLBDs

suject	query	Total Score	Query Cover	E-value	idengty	Acc.len
NcLBD53	AtLBD42	147	44%	4e-48	60.58%	202
NcLBD54	AtLBD41	273	100%	4e-96	53.17%	286
NcLBD55	AtLBD22	179	65%	2e-60	54.64%	251
NcLBD56	AtLBD40	228	65%	7e-80	70.39%	253
NcLBD57	AtLBD40	220	64%	1e-76	67.33%	257
NcLBD58	AtLBD23	85.5	64%	3e-27	53.25%	128
NcLBD59	AtLBD42	61.6	17%	1e-15	71.79%	182
NcLBD60	AtLBD22	196	83%	4e-66	48.93%	284
NcLBD61	AtLBD34	82.8	56%	3e-24	48.10%	257
NcLBD62	AtLBD3	171	64%	6e-59	72.90%	192
NcLBD63	AtLBD2	133	46%	4e-41	60.42%	435
NcLOB1	AtLBD42	211	59%	2e-73	71.53%	241
NcLOB2	AtLBD41	258	91%	9e-91	54.23%	266
