# Supplementary material for: Genome-Wide Identification and Expression Analysis of LBD Gene Family in Neolamarckia cadamba
Source: Int J Mol Sci. 2026 Jan 9;27(2):693. doi: 10.3390/ijms27020693 (PMC12841386; doi:10.3390/ijms27020693)
Supplement: Supplementary file 1 [file ijms-27-00693-s001.zip › Table S6 .pdf]

Table S6 The specific primers used in the qPCR

Gene Name	Primer sequence（5’-3’）	Amplicator length (bp)	Binding region	Efficiency (%)	Correlation Coefficient (R2)
Q-NcLBD4-F	GAGGAGCAACGAGAGGACAC	86	181-265nt	98.2	0.999
Q-NcLBD4-R	ACCAATGCAACCGTAAACCG				
Q-NcLBD9-F	GCACCCTATTTTGACCCGGA	88	82-167nt	96.3	0.986
Q-NcLBD9-R	AGCATCTTGGAGGCATTGCT				
Q-NcLBD10-F	CGGCCTCATGTCCTTCCTTT	90	468-555nt	92.5	0.983
Q-NcLBD10-R	CGTCCTCCCACAAGCTTCAT				
Q-NcLBD25-F	TTCGAAGCAGCTGGAAGGAC	130	205-335nt	101.2	0.999
Q-NcLBD25-R	TCCTGAATCGCCTGCAACTT				
Q-NcLBD37-F	AGTACGGTTTGGGTTGGGTC	95	490-583nt	98.0	0.982
Q-NcLBD37-R	CCGACTGGTGGCTCAAAGAT				
Q-NcLBD38-F	GGCCTGCTCTATTCCAGTCC	101	179-276nt	95.6	0.995
Q-NcLBD38-R	GTGCCAATTCCCCGTCCATA				

Note: E, amplification efficiency; R2,regression coefficient.
